# Characterization of Anisotropic Salt Weathering through Nondestructive Techniques Mapping Using a GIS Environment

**DOI:** 10.3390/s24092686

**Published:** 2024-04-24

**Authors:** Miguel Gomez-Heras, Laura López-González, María Teresa Gil-Muñoz, Cristina Cabello-Briones, David Benavente, Javier Martínez-Martínez

**Affiliations:** 1 Departamento de Geología y Geoquímica, Universidad Autónoma de Madrid, 28049 Madrid, Spain; 2 Independent Researcher, 28022 Madrid, Spain; aloplag@gmail.com; 3 Departamento de Pintura y Conservación-Restauración, Universidad Complutense de Madrid, 28040 Madrid, Spain; mariatgi@ucm.es; 4 Universidad Nacional de Educación a Distancia, 28015 Madrid, Spain; 5 Department of Earth and Environmental Sciences, University of Alicante, 03690 Alicante, Spain; david.benavente@ua.es; 6 Instituto Geológico y Minero de España (IGME, CSIC), Tres Cantos, 28760 Madrid, Spain; javier.martinez@igme.es

**Keywords:** stone decay, salt crystallization, anisotropic weathering, nondestructive testing, GIS, map algebra

## Abstract

Doctrinal texts on architectural heritage conservation emphasize the importance of fully understanding the structural and material characteristics and utilizing information systems. Photogrammetry allows for the generation of detailed, geo-referenced Digital Elevation Models of architectural elements at a low cost, while GIS software enables the addition of layers of material characteristic data to these models, creating different property maps that can be combined through map algebra. This paper presents the results of the mechanical characterization of materials and salt-related decay forms of the polygonal apse of the 13th-century monastery of Santa María de Bonaval (Guadalajara, Spain), which is primarily affected by salt crystallization. Rock strength is estimated using on-site nondestructive testing (ultrasound pulse velocity and Leeb hardness). They are mapped and combined through map algebra to derive a single mechanical soundness index (MSI) to determine whether the decay of the walls could be dependent on the orientation. The presented results show that salt decay in the building is anisotropic, with the south-facing side of the apse displaying an overall lower MSI than the others. The relative overheating of the south-facing side of the apse enhances the effect of salt crystallization, thereby promoting phase transitions between epsomite and hexahydrite.

## 1. Introduction

ICOMOS doctrinal texts and charters on architectural heritage conservation stress the importance of a full understanding of the structural and material characteristics [1] and the use of information systems and virtual presentation techniques [2,3,4,5].

Photogrammetry has been used extensively for creating virtual representation techniques for cultural heritage (e.g., [6,7,8,9,10]) and it is currently one of the most widespread methods to obtain 3D representations of heritage buildings. Multi-image photogrammetry or “Structure from Motion” (SfM), as it is sometimes called, is preferred to other representation techniques, such as LiDAR, as it can generate high-accuracy point clouds at a lower cost with more flexibility of scale [11]. These characteristics make photogrammetry particularly useful for contorted surfaces with many occlusions [12,13]. Photogrammetric models have been combined with GIS in different ways, mostly using GIS in the usual way as a tool to manage information within a territory [14,15,16]. However, other applications have used Digital Elevation Models (DEM) obtained from multi-image photogrammetry of architectural elements, considering walls as horizontal topographic surfaces. Therefore, by setting an arbitrary vertical plane as a horizontal datum, all the values in the model have a positive elevation. Material properties data can be superimposed on these “architectural” DEMs in a GIS environment, creating a high-detail “architectural GIS” [9,17]. This allows for the consideration of historic building elements as topographic surfaces in which analytical measurements of material properties can be represented and managed as “geographical” data within a GIS. This includes combining measurements, carrying out data operations, and even integrating multispectral imagery [9,17,18].

Mapping material properties is the best way to find spatial trends and determine if decay acts directionally. Non-uniform decay patterns can be the result of climatic weathering agents (like insolation, wind, and consequently driven rain), acting in preferential directions [19,20] either directly or through their influence on other major decay agents, such as salt crystallization. Thus, assessing the different weathering rates of the different aspects of a building offers information on past and present environmental anisotropy.

Nondestructive testing is the most appropriate procedure to gather enough data on material properties over the whole extent of a heritage building for the purpose of mapping. Ultrasonic pulse velocity (UPV) and Leeb hardness (LH) are among the most commonly used techniques to nondestructively estimate the compressive strength of materials in heritage contexts [21,22]. UPV refers to the results obtained using portable equipment that measures the time spent by an ultrasonic wave to travel a specific distance through a material. As P-waves are the first arrivals recorded in a non-polarized ultrasonic wave, and in most cases, UPV denotes the P-wave propagation velocity. LH is calculated from the ratio between the impact and rebound velocities of a 3 mm spherical impacting tip. The small size of the tip and low impact energy (11.5 N·mm for a D-probe) allow this device to perform minimally destructive rebound hardness.

This paper presents the utilization of GIS for mapping nondestructive techniques, particularly those pertaining to estimators of rock strength. This demonstrates how these results can be integrated into a unified map to assess whether the decay of walls induced by salt crystallization and phase changes depends on the orientation. To do so, the polygonal apse of a 13th-century monastery located in Central Spain was selected.

## 2. Materials and Methods

### 2.1. The Monastery of Santa María de Bonaval

**Figure 1:**
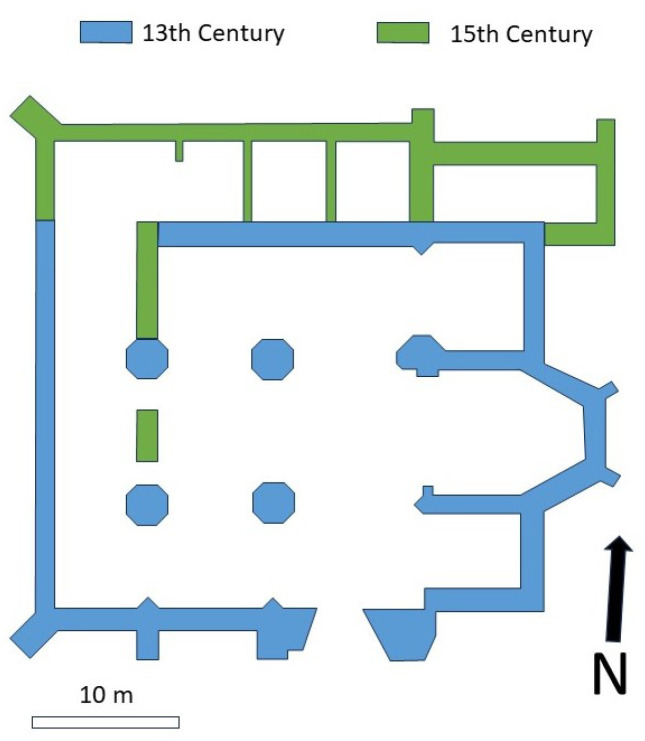
Schematic plan of the preserved ruins showing the different building phases.

The Monastery of Santa María de Bonaval is a Cistercian Monastery located in the municipality of Retiendas (Guadalajara) in Central Spain, around 80 km NNE from Madrid. The oldest parts of this Monastery date back to the 13th century, and they were built in Gothic style. Only a portion of the original building has been preserved, primarily consisting of the church’s apses, transepts, southern naves, and an attached sacristy (Figure 1).

The building underwent extensive modifications of its internal structure during the 15th century, readapting parts of the church into other monastic facilities whose remnants are visible in the northern parts of the building. The present layout is slightly rectangular, measuring 42 m in length and 37 m in width, featuring three naves and two sections. The southern wall, transept, and apse are externally reinforced with buttresses. The head comprises three apses, with the central one being polygonal with three sides and the lateral ones being rectangular. The building was abandoned after several confiscations of the goods of the Catholic Church by the Spanish government during the 19th century. The land where the monastery is located was transferred to several private owners, and the building evolved into the current ruins. The ruins were restored in 2018 and they are now visitable.

**Figure 2:**
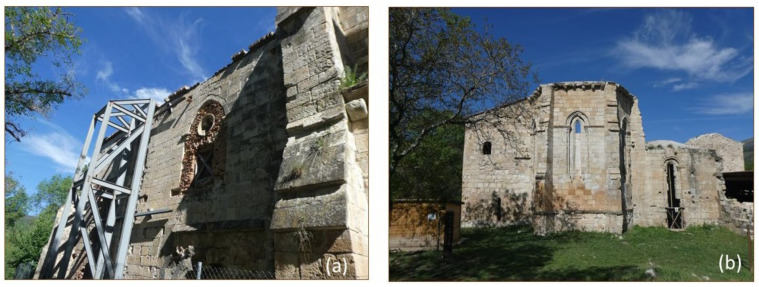
Views of the monastery of Santa María de Bonaval from the south (**a**) and the east (**b**). The building shows a marked anisotropy of decay: the south and west sections of the building show more overall structural failure. The south and east sections show the largest proportion of salt-related decay forms. Nevertheless, the east part of the building is the best-preserved area.

**Figure 3:**
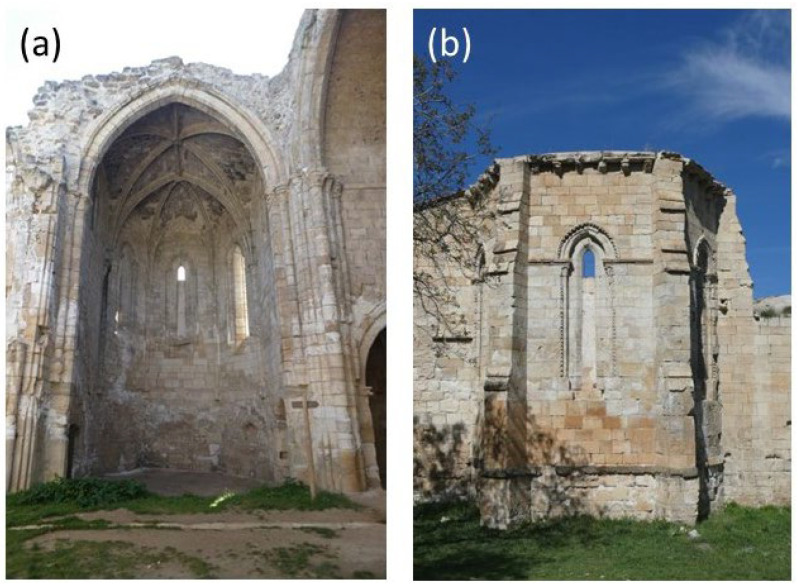
Pictures of the main apse of the monastery’s church from the inside (**a**) and the outside (**b**).

The building is located around 100 m to the south of the Jarama River and 100 m to the north of a hill within the so-called Bustar’s ravine. In addition to large-scale structural damage (Figure 2), the location of the monastery near the floodplain in an agricultural area promotes salt crystallization as the most extensive decay process involved, particularly in the inside part of the main apse of the church (Figure 3a). Nevertheless, the external side of the main apse is the best-preserved area of the building (Figure 3b).

The polygonal apse on the east side of the building consists of three walls separated by buttresses: while the central one faces completely to the east, the other ones are tilted to the north and to the south, respectively. This change in orientation modifies the amount of insolation received, and therefore, it is expected to have a significant influence on salt weathering. Therefore, this area was selected to map the results of nondestructive tests to determine if mechanical strength was dependent on the orientation as a consequence of the anisotropy of salt weathering processes.

### 2.2. Material Characterization

A sample block of the material was obtained. Rock specimens were cut from this block into regular-sized samples of approximately 1 cm^3^ to measure the connected porosity and pore-size distribution using mercury intrusion porosimetry. Also, six 40 mm cubes were cut to measure the capillary coefficient and uniaxial compressive strength.

The pore structure characterization was described in terms of porosity and pore-size distribution using mercury intrusion porosimetry (MIP). A PoreMaster 60 GT (Quantachrome Instruments) mercury porosimeter was used to detect pores in the pore radius interval of 0.002–200 μm. Uniaxial compressive strength was characterized following the EN-1926 standardized test [23]. The water absorption by capillarity tests was carried out using a continuous data-recording device [24]. The results were plotted as absorbed water per area of the sample throughout imbibition versus the square root of time. The slope of the curve during capillary absorption is the capillary absorption coefficient (C).

The mineralogy of salt efflorescences was analyzed by powder X-ray diffraction (XRD) on a Bruker D8-Advance diffractometer with mirror Goebel (non-planar samples) using the CuKa radiation at 40 kV and 40 mA, 2θ: 3–60°, step size of 0.05°, and scan step of 3 s. Data were collected and interpreted using the XPowder software (https://www.xpowder.com/) package [25]. The qualitative search–matching procedure was based on the ICDD-PDF2 database.

Ultrasonic pulse velocity and Leeb rebound hardness were measured for the two cut rock blocks. A Proceq Pundit lab portable ultrasonic test equipment, with 54 kHz P-polarized transducers, was used. Measurements were made using the direct mode of transmission (transducers were applied on opposite parallel sides of the block). A viscoelastic solid couplant (plasticine) was used to ensure good coupling between the transducers and the stone blocks. Ten measurements were taken overall. Twenty-five measurements of Leeb rebound hardness were made with an Equotip Piccolo with a D-probe (hence, the values obtained are Leeb hardness with D-probe—LHD).

### 2.3. On-Site Nondestructive Testing

Ultrasonic pulse velocity (UPV) and Leeb rebound hardness (LHD) tests were used to nondestructively determine the mechanical strength of the lower section of the apse’s walls. The same Proceq Pundit lab portable ultrasonic test equipment, with 54 kHz P-polarized transducers, was used. Measurements on the building blocks were made using the indirect mode of transmission (both transducers were applied on the same surface) keeping the distance between the centers of the transducers, covered with a viscoelastic couplant, at a constant distance of 15 cm. Three measurements were made for each stone block (horizontal, vertical, and diagonal), and the average of the three values was used for mapping. Leeb rebound hardness was measured using an Equotip Piccolo instrument with a D-probe. Five measurements were recorded in each block and the average of these five values was used for mapping.

Additionally, environmental conditions were measured at each wall of the apse, and infrared images were taken using a FLIR T1020 camera equipped with a 36 mm lens to visualize the variations in apparent surface temperature in the different orientations of the apse’s walls. Images were taken as single passive thermography shots several times until the local solar midday of the survey date (26 April 2023). IR images were taken from the east, so they included three sections of the apse in a single shot. IR images were corrected using FLIR tools software. Atmospheric correction was performed, but emissivity was fixed at 0.95 for all images, as the main interest was to obtain an approximation of the differences in temperature of the different walls made of the same material.

On-site observations of rock weathering patterns were carried out using a portable handheld digital microscope (DinoLite WF4115ZTL) at up to 140× magnification.

### 2.4. Photogrammetry and Data Mapping

**Figure 4:**
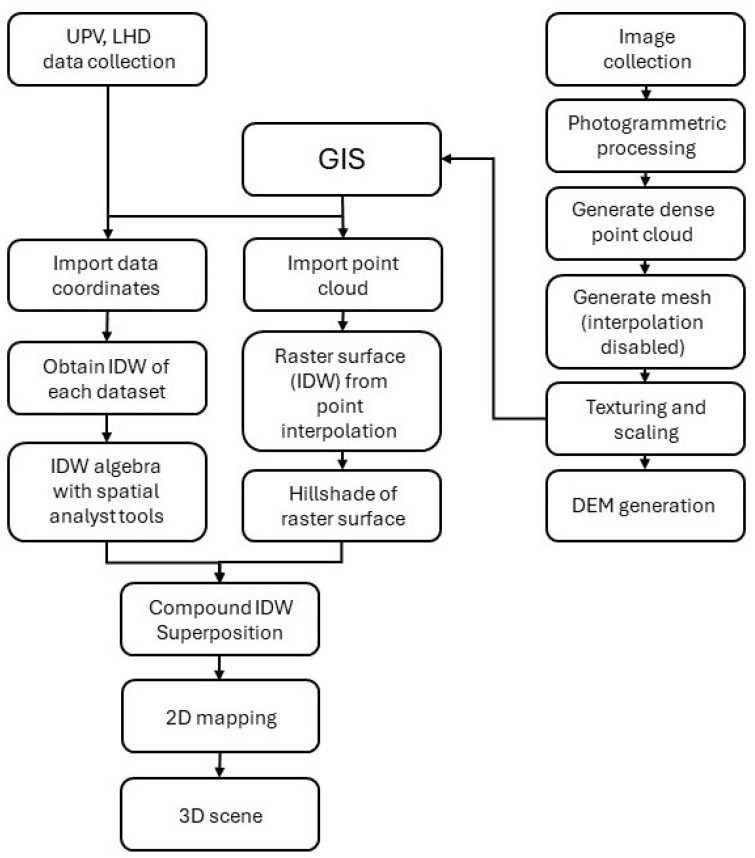
Methodological workflow of photogrammetry and data mapping.

To capture a three-dimensional Digital Elevation Model (DEM) of the apse’s walls, multi-image photogrammetry was used. This technique involves capturing rectified photographs of the object from various angles and processing them with specialized software (Agisoft Metashape Professional 1.8.5). Figure 4 shows a simplified workflow of the methodology in relation to photogrammetry and data mapping.

**Figure 5:**
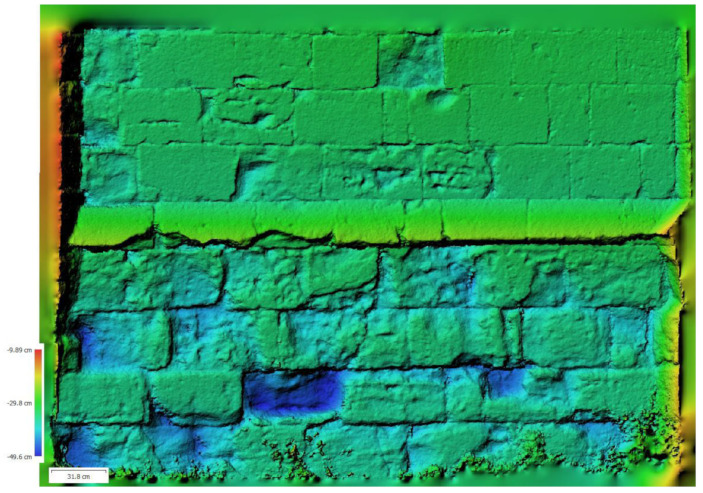
Colored DEM of the SE wall of the apse with a scale showing receded areas in blue. Distances were measured from an arbitrary datum placed in front of the DEM.

The Agisoft Metashape Professional program is a photogrammetry software that generates 3D point clouds from a series of ordered photographic images. Photographs must be taken as parallel to the façade as possible, overlapping each photo with the previous ones with reference points between them. In this study, a sequence of thirty photographs was taken using a Pentax KD10 camera fitted with a 10.2 Mpx CCD sensor (focal length 18 and ISO100). Photographs were taken parallel to the walls, maintaining a constant distance of approximately 3 m. Although the software has a certain tolerance that allows overlapping images taken at different heights, photographs were taken while maintaining a constant height (approximately 1.5 m from the ground due to the uneven terrain). The focus angle was varied to ensure sufficient overlap between successive photographs, with the overlap coinciding with the center of each image. Twelve eight-bit coded targets were strategically positioned across the apse to establish the reference axes, resulting in a comprehensive point cloud of 23,439 points. Each wall was then individualized with a different orientation (east, south, and west), leading to a 3D model comprising 1,535,019 faces and 9,524,779 points. A DEM was subsequently generated from this point cloud (Figure 5). The DEM is a raster surface, where each cell of a matrix corresponds to a 2.9 mm cell with X, Y, and Z values (Z values are measured from an arbitrary datum placed in front of the DEM). For a better visualization, Figure 5 shows a layer that simulates shading instead of seeing the raster cells. This allows better visualization of the topography of the surface than with the point cloud from which it originates.

The processed raster layers from the scaled and georeferenced point cloud, using ArcGIS 10.2 software, yielded a topographic surface. The point cloud data were converted into a shapefile (.shp) vectorial digital storage file, providing indexed spatial element identifiers (FID) for each point. Inverse distance-weighted interpolation of the .shp file data facilitated the creation of a highly accurate raster surface, including gap filling within the point cloud. The raster layer was hill-shaded to enhance texture visualization, revealing details not visible to the naked eye or in photographs. The different orientations of the apse (east, south, and west) were modeled separately due to the current software limitations in handling elements that “bend” in space or overlap on the same plane.

For each FID (Feature ID), in situ measurement data were introduced to generate data maps, creating distinct raster layers for each set of measurements (UPV and LHD). As both UPV and LHD relate to mechanical strength, a “mechanical soundness index” (MSI) was devised to merge both sets of data into one through map algebra:

(1) $$MSI=\left(\frac{UPV\;(\frac{m}{s})}{1000}+\frac{LHD}{100}\right)$$

This index sets the UPV and LHD in the same order of magnitude, so they contribute the same to the sum of both values. The rationale for combining both values to derive a comprehensive measure of soundness is grounded in the fact that, while both techniques are directly linked to compressive strength, they exhibit sensitivity to distinct rock parameters. LHD offers a more accurate indication of material strength at a smaller scale. In contrast, UPV provides insights into larger-scale discontinuities, influenced by a greater mass of rock compared to the LHD rebound number. This index ensures a more holistic assessment of the material’s overall soundness by capturing information at both small and large scales. Hence, the higher the MSI, the higher the material’s overall soundness, considering both small and large scales.

## 3. Results

**Figure 6:**
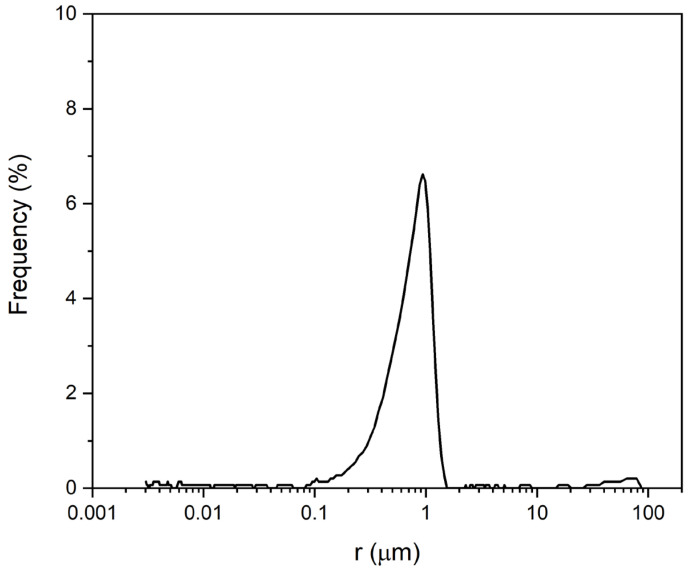
Pore-size distribution of the Santa María de Bonaval dolostone.

The monastery is built with a homogeneous dolostone with 29.1% connected porosity, which is similar to the open porosity measured in the 4 cm cubic samples (Table 1). The pore-size distribution of the building stone shows a main pore population that ranges from 0.1 to 1 μm (Figure 6), which is related to its well-sorted texture.

The analyzed dolostone exhibits a low resistance (Table 1) according to Deere and Miller [26], although it is higher than other carbonatic porous building stones widely used in the built heritage [27]. Consequently, the pore structure and the relatively low mechanical strength of these building materials make them susceptible to stone deterioration. The ultrasonic pulse velocity of the cut rock blocks is 3800 ± 100 m/s and the Leeb rebound hardness is 390 ± 20.

The mineral composition of the salt efflorescences shows the abundant presence of epsomite (MgSO_4_·7H_2_O) and, in minor amounts, niter (KNO_3_). The origin of the epsomite is linked to the alteration of the Mg-bearing dolostone, whereas the presence of niter can be attributed to the proximity to the floodplain in an agricultural area.

**Figure 7:**
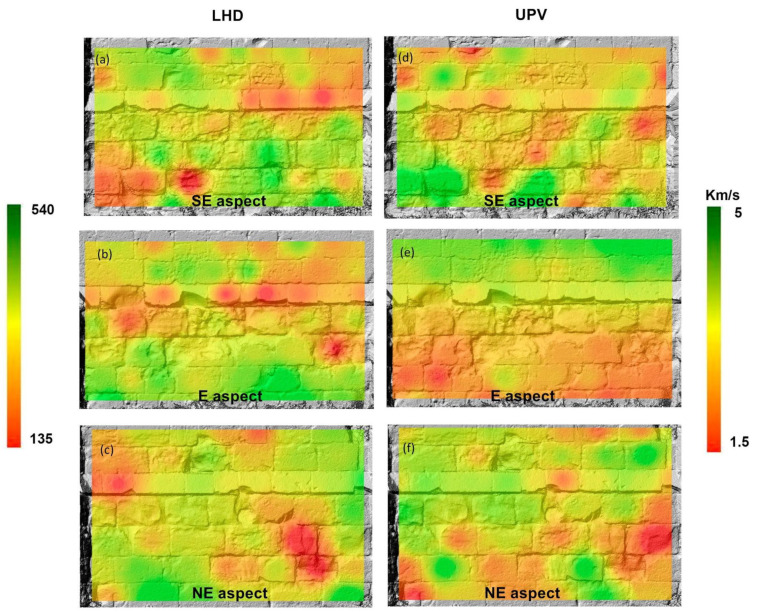
LHD (**a**–**c**) and UPV (**d**–**f**) maps obtained from GIS of each of the surveyed walls expressed on a color scale from the minimum to the maximum values obtained during the survey. LHD is a non-dimensional magnitude; hence, the scale does not have units.

Figure 7 shows the cartographic results of LHD and UPV overlapping the raster layers. Although the representation on paper makes it look like a static 2D representation, it must be noted that these are dynamic 3D models, as can be seen in the example in Supplementary Material Figure S1.

Both sets of results demonstrate how, although both parameters link directly to compressive strength, they yield different attributes of what causes the diminishing of strength. However, in either case, a decay pattern emerges in both the external walls of the apse of strips, seemingly converging to the central (east) wall.

**Figure 8:**
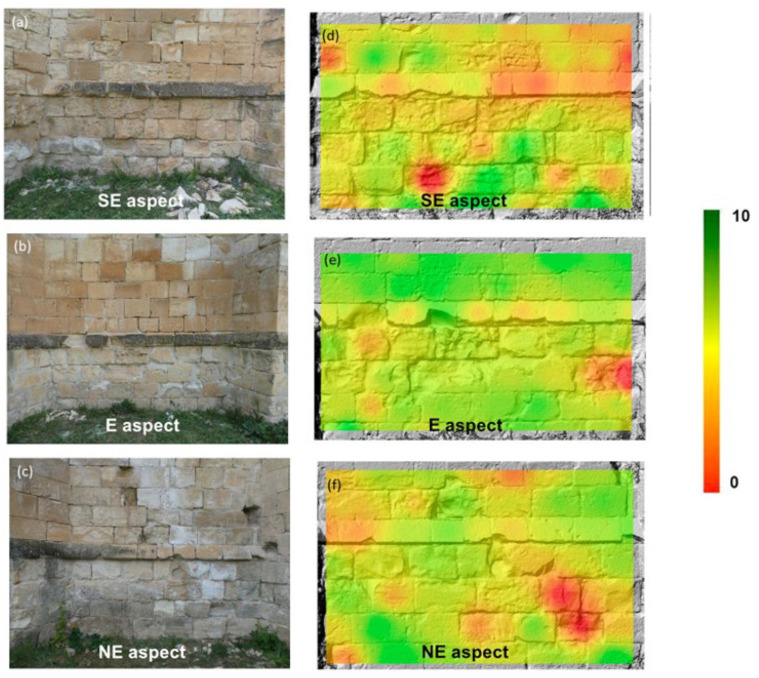
Pictures of the three walls of the studied apse correspond to the different aspects (**a**–**c**) and the equivalent MSI maps (**d**–**f**) obtained from the GIS map algebra. MSI is non-dimensional.

Figure 8 displays the mechanical soundness index map (Figure 8d–f) obtained using map algebra within a GIS that used DEMs of each section of the apse as a topographic base. The lower the MSI, the lower the combined normalized value of UPV and HLD. Therefore, the most decayed areas had lower values of the MSI.

MSI combines the normalized measurements of UPV and LHD and gives them the same weight. The results of MSI mapping make the decay patterns converging to the central part of the apse more noticeable, which, as shown in the photographs, correspond to stripes where salt efflorescences are visible. The MSI map also shows that the SE wall of the apse displays the most extensive strength loss.

**Figure 9:**
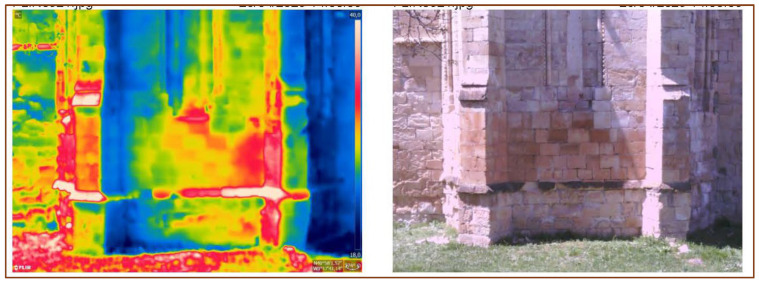
IR (**left**) and visible (**right**) images of the apse at local midday close to the Spring equinox.

Figure 9 shows a representative IR and visible image of the apse taken at local midday from the east. At midday, the south-facing areas of the apse show a significantly higher surface temperature (average temperature of around 34 °C) in comparison to the north-facing areas of the apse (average temperature of around 21 °C). The environmental conditions at the time were 20 °C and 50% RH.

## 4. Discussion

The results show an overall deterioration of the apse of the Monastery of Santa María de Bonaval, with extensive areas of all surveyed walls displaying values of ultrasonic pulse velocity and Leeb rebound hardness, which are lower than those obtained from unaltered cut rock blocks. The overall decay of the south-facing areas of the apse is significantly higher, as shown in the MSI map.

**Figure 10:**
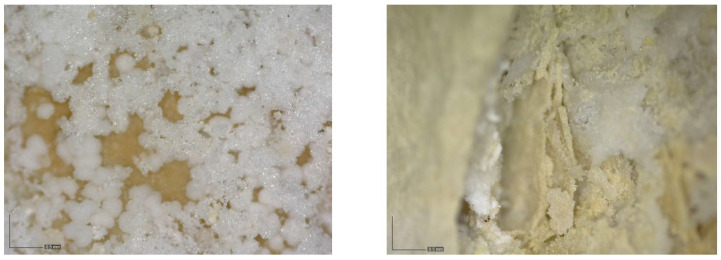
Photomicrographs obtained with a portable digital microscope showing powdery efflorescences (**left**) and subefflorescences associated with flacks and scales (**right**).

The main decay factor of the walls is salt crystallization, as shown by the efflorescences and subefflorescences throughout the walls (Figure 10). Salts appear as powdery aggregates, with slight skeletal development. Crystallization in the rock surface and in subsurface layers causes granular disintegration and scaling of the rock (according to the terminology of the ICOMOS-ISCS glossary [28]). The place where the salt crystallizes is controlled by both the hydric properties of the stone and the evaporation conditions of the salt solution (ultimately controlled by the T-RH conditions). The formation of both efflorescences and subflorescences and their relationship with different decay patterns have been extensively studied by [29]. In the specific case of magnesium sulfate solutions, Sato et al. [29] demonstrated that this salt tends to preferentially form subflorescences, unlike other salts such as NaCl or Na_2_SO_4_, which are mostly related to efflorescences.

Capillary transport through the dolostone is the main mechanism by which saline solutions access the lower parts of the building. According to the pore structure of the dolostone (pore size and connected porosity), capillary movement is active. Thus, the capillarity absorption rate of the porous biocalcarenite, quantified using the capillary coefficient, presents relatively high values (Table 1) compared with other carbonatic porous building materials [30].

Moreover, the susceptibility of porous materials to salt weathering is linked to both their pore structure and mechanical properties. Salt crystallization typically occurs within the 0.1–10 µm pore size range, demanding high supersaturation for percolation below 0.1 µm—conditions uncommon in the built environment. Larger pores (>10 µm) allow salt permeation without imposing significant crystallization pressure on the pore walls, and building materials contribute significantly to the 0.1–10 µm range and affect porosity. Furthermore, the effectiveness of the crystallization pressure within the material is conditional on the rock’s strength, representing its resistance to the mechanical effects of salt crystallization.

**Figure 11:**
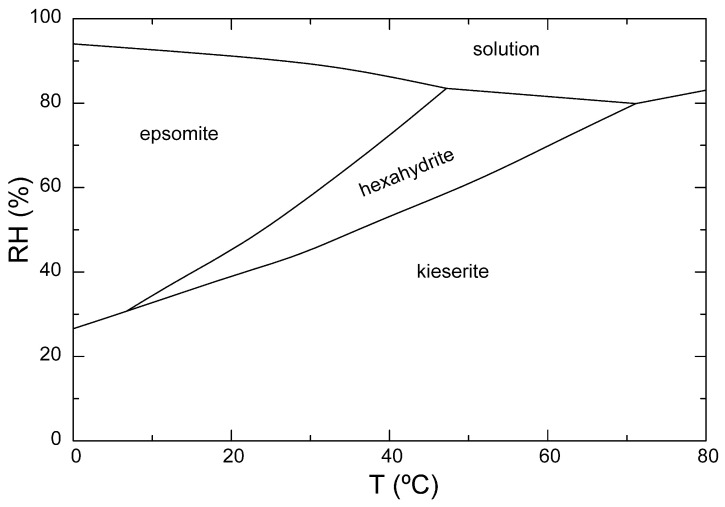
Phase diagram of the MgSO_4_ system (modified from [37]).

Epsomite (MgSO_4_·7H_2_O) is the main phase observed in the efflorescences. The MgSO_4_ mineral group presents different hydrated mineral phases such as epsomite, hexahydrite (MgSO_4_·6H_2_O), and kieserite (MgSO_4_·H_2_O), which are considered to be one of the most aggressive salt systems in comparison to other salts, such as nitrates and chlorides [31,32]. In particular, the dissolution of the less hydrated form (hexahydrite), which is followed by the precipitation of the hydrated salt (epsomite), is the most aggressive weathering mechanism for these types of salts. This precipitation pathway occurs by crossing the phase boundary (Figure 11), and it can generate enough stress to damage the stone [33,34]. In particular, the transition between epsomite and hexahydrite takes place under common environmental conditions, and daily and seasonal T and RH variations have been shown to be factors in this phase transition [35].

As discussed above, the microstructure of the porous dolostone favors the capillary transport of salts and the effectiveness of salt crystallization, explaining the rapid and severe damage due to salt crystallization caused by rising dampness. For example, considering the environmental conditions and surface temperatures when the thermal image in Figure 9 was obtained, the north-facing façade of the apse (average temperature of around 21 °C) falls within the epsomite field for a 50% RH, while the south-facing façade of the apse (average temperature of around 34 °C) falls within the hexahydrite field for the samee RH. The relative overheating of the south-facing side of the apse could mean that it undergoes more epsomite–hexahydrite phase transitions as a consequence, multiplying the destructive effects of salt crystallization in this part of the apse, which is consistent with the overall reduced MSI map (Figure 8).

Solar radiation significantly influences the microenvironment around buildings and monuments, as demonstrated in previous studies [20]. Therefore, the weathering processes related to solar radiation will vary in intensity depending on the façade orientation, generating an anisotropic decay. The cycles of solar radiation range from large yearly and daily cycles of heating and cooling to small-scale fluctuations due to clouds or surrounding elements blocking temporal sunrays [36]. In the northern hemisphere, these cycles will be prevalent in southern orientations, creating an anisotropic microenvironment at the building scale. This is shown by the differences between the air temperature and rock surface temperature. The northern façades of the Santa María de Bonaval Monastery show apparent rock surface temperatures, which are like air temperatures (20–21 °C). However, rock surface temperatures in the southern façades exceed the air temperature by 14 °C.

## 5. Conclusions

This paper demonstrates how GIS software facilitates the manipulation of multiple layers of rock strength estimation data, enabling their integration into maps that combine various sets of results reflecting different factors contributing to strength reduction. In this study, a mechanical soundness index was introduced to standardize the values of the ultrasound velocity and Leeb hardness, ensuring equal contributions to the combined value.

The resulting maps emphasize the influence of salt-related weathering on a 13th-century monastery constructed with dolostone, which is heavily affected by salt efflorescences. Analysis of the efflorescences revealed epsomite as the predominant mineral phase. Environmental conditions, such as temperature and humidity, induce phase transitions between epsomite and hexahydrite, exacerbating the detrimental effects of salts on historic structures.

Infrared imaging data showed significantly higher temperatures on the south-facing side of the apse compared to other aspects throughout the day, promoting more frequent epsomite–hexahydrite phase transitions. This environmental variability contributes to the anisotropic distribution of salt weathering, resulting in uneven decay patterns across the building.

The comprehension of the directional aspects of decay and weathering processes remains significantly understudied, despite their crucial role in scaling climate effects down to the building level. Understanding anisotropic decay at the building scale is imperative to plan more effectively and implement preventive measures to minimize climate-induced decay in both current and future scenarios, especially in the context of the ongoing climate emergency.

## Figures and Tables

**Table 1:** Uniaxial compressive strength (UCS), open porosity (P_O_), and capillary absorption coefficient (C) of the Santa Maria de Bonaval dolostone.

N Sample	UCS (MPa)	P_O_ (%)	C (g/m^2^s^0.5^)
1	38.86	32.31	20.61
2	49.71	29.94	19.48
3	51.18	29.59	20.30
4	42.90	30.92	20.09
5	39.41	31.38	20.22
6	47.26	29.23	18.51
**Mean**	**44.89**	**30.56**	**19.87**
**St. Dev**	**5.27**	**1.18**	**0.76**

## Data Availability

Data are available upon request by the corresponding authors.

## Supplementary Materials

The following supporting information can be downloaded at: https://www.mdpi.com/article/10.3390/s24092686/s1, Figure S1: animation showing the 3D characteristics of one of the MSI maps.
